# Isolation frequency of *Candida* present on the surfaces of mobile phones and handsx

**DOI:** 10.1186/s12879-016-1577-0

**Published:** 2016-06-01

**Authors:** Anna Kordecka, Elżbieta Krajewska-Kułak, Cecylia Łukaszuk, Bogumiła Kraszyńska, Wojciech Kułak

**Affiliations:** Department of Anesthesiology and Intensive Therapy, Medical University of Białystok, Skłodowskiej 7a, 15-096 Białystok, Poland; Department of Integrated Medical Care, Medical University of Białystok, Białystok, Poland; Department of Pediatric Rehabilitation, Medical University of Białystok, Białystok, Poland

**Keywords:** Hands, Mobile phone, Candida

## Abstract

**Background:**

It is known that mobile phones may play a role in microorganism transmission. The aim of this study was to analyze the relationship between the number of *Candida* genera/species isolated from samples collected from the surfaces of mobile phones and the hands of the staff as well as the preferred health-related behavior.

**Methods:**

The mycological evaluation included 175 mobile telephones and the hands of staff members at the University Hospital in Białystok, Poland. We used the Count-Tact^TM^ applicator, with CandiSelect (Bio-Rad). Self-administered questionnaire was used to gather data on mobile phones disinfection practices. Assessment of the preferred health-related behavior was based on The Multidemensional Health Locus of Control Scale (MHLC).

**Results:**

Out of 175 mobile phones, 131 (74.9 %) were colonized. *Candida glabrata*, *C. albicans* and *C.krusei* were isolated more frequently from the hand as well as phone surface. The mean number of *Candida* colonies was higher in samples collected from hand surfaces than mobile phone surfaces. No significant correlation was found between the preferred health-related behavior and the frequency of washing hands, the way of using a mobile phone, the number of colonies or the isolation frequency for the fungi collected from the surface of the phones and hands of their owners. Only 19.4 % of the participants cleaned the surface of their phones.

**Conclusion:**

The prevalence of mobile phone contamination by *Candida* is high in the University Hospital in Białystok, Poland. *Candida albicans*, *C. glabrata,* and *C. krusei* were the dominant species in the samples collected from mobile phones and hands. These results pose the need to develop guidelines for mobile phone disinfection.

## Background

The estimated number of bacteria found on the hands of medical personnel ranges between 3.9 x 10^4^ and 4.6 x 10^6^ bacteria/cm^2^, and it increases with increasing duration of the performed clinical procedures, at a rate of 16 cells per minute [1–3]. The risk of developing the infection is more in health care settings. The contaminated hands of medical staff play a major role in spreading infections in healthcare settings. Hand hygiene is one of the most important preventive interventions against the spread of infections in healthcare settings [4]. Mobile phones have become increasingly integrated into the practice of doctors and allied medical professionals.

Mark et al. [5] investigated the level of contamination on phones used in surgical wards. Included in the study were 150 healthcare workers’ mobile telephones. Bacterial contamination was found on 60 % of the phones. In total, 88 % of the respondents used their phones at work, and 55 % used them for clinical purposes.

A study by Ülger et al. [6] in Turkey, which examined 200 mobile phones belonging to operating-room and intensive-care personnel, found different species of bacteria, including G (−) (31.3 %) and of *Staphylococcus aureus* (5.2 %), isolated from samples obtained from 94.5 % of physicians’ phones.

Nearly 35 % of mobile phones were culture-positive for two types of bacteria, and more than 11 % were inhabited by three or more strains. It was also shown that only 10 % of medical personnel regularly cleaned their mobile phones.

Kilic et al. [7] studied 106 cell phones belonging to health care providers, and from samples obtained from 61.3 % isolated mainly *Staphylococcus epidermidis*, *Staphylococcus aureus*, *Bacillus sp.*, *Corynebacterium sp.* and *Escherichia coli*.

In the past years, a significantly increased frequency of invasive fungal infections has been repeatedly reported worldwide [8], which are a serious epidemiological, clinical, diagnostic, and therapeutic problem, as well as an increased role of fungi in the spread of nosocomial infections [8, 9]. *Candida* species is the fourth most common source of hospital-acquired infections. Yeasts are part of our normal micro-flora and invasive infections arise only when barrier leakage or impaired immune function occurs. Delfino et al. [9] in their retrospective study (2008–2012) confirmed that candidaemia in intensive care units patients is caused predominantly by strains colonizing healthcare workers. Approximately, 39 % (50/129) of healthcare workers were positive for yeasts and among 77 different fungal isolates recovered, *C. parapsilosis* was the most frequent (44/77; 57 %). Twenty-seven diverse genotypes were obtained by microsatellite analysis of 42 selected blood and hand isolates. Most of the isolates from the hands showed a new, unrelated genotype, whereas a particular group of closely related genotypes prevailed in the blood samples.

The reports on fungi isolated from the surfaces of both mobile phones and the hands of their owners are still sparse in the available medical literature [10].

Özkan and Sülün, [9] examined 50 mobile phones used by Health Services Vocational School students. A total of 24 different microfungal species were obtained belonging to *Alternaria, Aspergillus, Cladosporium, Geotrichum, Penicillium, Phoma, Rhinocladiella, Scopulariopsis, Trichoderma, and Trichophyton genera.*

The aim of the study was to analyze the relationship between the number and the genera/species of *Candida* isolated from samples collected from the surfaces of mobile telephones and the hands of their owners as well as the preferred health-related behavior and hand washing frequency.

## Methods

A total of 175 mobile phones and hands of students (*n =* 142) and professors (*n =* 33) of the Medical University Hospital of Białystok were included in the mycological evaluation.

### Data collection and laboratory methods

A self-administered questionnaire was used to collect data on the participants’ demographics, which included age, gender, knowledge about mobile contamination, and the duration of the participants’ mobile phone use. Other questions regarding their use of mobile phones at work and their perception of the potential role of clinicians’ mobile phones in spreading infections in hospital settings also appeared on the questionnaire.

The questionnaire also included questions on mobile phone hygiene practices; including the frequency of mobile phone disinfection and the disinfectant which clinicians used to clean their mobiles. Assessment of the preferred health-related behavior was based on The Multidemensional Health Locus of Control Scale (MHLC) - version B - by Wallston K.A., Wallston B.S., Devellis R., Polish adaptation by Juczyński [11].

Biological monitoring of mobile phone and hand surface contamination was performed with Count-TactTM applicator using Count-Tact plates (bioMerieux) containing a medium complying with the requirements of the Draft European Standard CEN/TC 243/WG2. CandiSelect (Bio-Rad) was used to identify yeast-like fungi*.* The mycological procedures were in accordance with the manufacturer's instructions. In case of mobile phones with covers, the sample was taken from the outer surfaces of the cover in addition to the screen of the mobile phone.

### Statistical analysis

Statistics were calculated using Statistica 10.0 PL (StatSoft, Tulsa, OK). Selected numerical characteristics of the evaluated parameters such as: the arithmetic mean; median; the highest (maximum) and the lowest (minimum) values; standard deviation (*s*), which is a measure of “average” deviation from the mean value; 25th and 75th percentile, first and third quartiles; Spearman rank correlation coefficient; and Wilcoxon test were used for statistical analysis. Differences were considered statistically significant when *p <* 0.05.

## Results

Most respondents (85.1 %) owned one mobile telephone, 13.7 % of the respondents had two phones, and 1.1 % owned more than two. The average duration for mobile phone use was 9.4 years, with the last phone being used for an average of 1.9 years.

The majority of respondents (81.1 %) declared that they had heard that the mobile phone surface may be inhabited by microorganisms, including bacteria (80 %), fungi (60.6 %), and viruses (28.6 %). A total of 10.2 % of the respondents had difficulty answering the question. Every third respondent (32.6 %) did not clean their phone surfaces, whereas every fifth respondent (19.4 %) did.

The majority of respondents who cleaned their phones occasionally disinfected the phone surface (46.9 %), while others did so once a month (13.3), once a week (7.4 %), every day (1.7 %), or did not specify how often (4 %). Virtually all respondents (96.6 %) had no knowledge of mobile phone disinfecting machines. Only 3.4 % of respondents were familiar with this fact. More than half of the respondents (53.1 %) expressed their willingness to have their phones professionally cleaned, nearly every fifth respondent (18.9 %) was not interested in such an option, and 28 % were undeclared.

Phones were most frequently kept in a shoulder bag (54.9 %). A total of 43.4 % of the respondents kept their phones in their pockets, while 14.3 % kept them in a desk or cabinet.

When moving around, the respondents usually kept their phones in a bag (50.3 %), pocket (44 %), or hand (one in ten respondents - 10.3 %).

Most respondents (about two-thirds; 64.6 %) always remembered to wash their hands in the morning (after getting up) and before bedtime. Most of them (94.4 %) had a habit of washing their hands after going to the toilet. The majority (69.7 %) remembered to wash their hands before preparing meals, after contact with a patient (68 %), after returning home (57.1 %), after contact with pets (54.3 %), and before having a meal (53.7 %). Removing jewelry (18.9 %) and watches (25.1 %) was the least common habit prior to washing hands. Most respondents used soap in special dispensers (53.1 %) while washing their hands, and every third respondent washed their hands using a bar of soap interchangeably with soap in a dispenser (32.6 %). Bars of soap were used by 23.4 % of respondents, whereas 0.6 % used water only. Two-thirds of respondents (65.1 %) used tepid water to wash their hands. Others used hot water (17.7 %), water of varying temperatures (16.6 %), or cold water (2.3 %). Most respondents used fabric towels (56 %), while only one in six respondents (16 %) used disposable towels. Fabric towels were used interchangeably with disposable towels by 30.0 % of respondents, while 5.1 % preferred driers.

As can be seen from the data in Table 1, *C. glabrata* dominated among the fungi identified in the collected samples, however, *C.albicans* and *C.krusei* were also common. These three species were found on over half of the respondents, both on their hands and their phone surfaces. In contrast, *C. tropicalis* and the genus *C. species* occurred sporadically.

**Table 1:** Species/genera of *Candida* isolated from the samples collected from hand and cell phone surfaces

Species/genera of *Candida*	The occurrence of fungal strains in samples taken from			
	hand surface		telephone surface	
	Number	Percent^a)^	Number	Percent^a)^
*Candida glabrata*	156	89.1 %	131	74.9 %
*Candida albicans*	146	83.4 %	114	65.1 %
*Candida krusei*	122	69.7 %	95	54.3 %
*Candida tropicalis*	9	5.1 %	11	6.3 %
*Candida species*	1	0.6 %	0	0.0 %
Non-*Candida*	1	0.6 %	10	5.7 %

^a)^ Sums do not have to add up to 100 %, as any number of response options could be chosen

In the next stage of the study, a correlation between the occurrence of the same genera/species of fungal strains on hand and phone surfaces was investigated.

For this purpose, data on the presence of *Candida* of a certain genus/species on hand and phone surfaces was summarized in the form of a cross-table with percentages displayed in the column-and-row structure (Table 1).

The chi-square test for independence was performed to assess whether there is a relationship between the presence of certain species/genus on hand and phone surfaces. The results are shown in the table headings. The sporadically occurring *Candida species* was not included in the analysis.

We found a clear correlation between the co-existence of *C. albicans* in samples collected from hand and mobile phone surfaces.

Analysis, for example, of column percentages allows to conclude that *C. albicans* was present on the hands of 95 % of 114 subjects who had this species isolated from their phone surfaces. *C. albicans* was identified in 74 % of 146 subjects who were culture-positive for *C. albicans* on their hand and phone surfaces (Table 2). The obtained results clearly indicate that lack of adequate hand hygiene resulted in the presence of fungi on mobile phones.

**Table 2:** Correlation between the co-existence of *Candida* strains in samples collected from hand and mobile phone surfaces

Phone	Hand		Total
	no	yes	
Candida albicans (*p =* 0.0000)			
no	23 (38 %_→_/79 %_↓_)	38 (62 %_→_/26 %_↓_)	61
yes	6 (5 %_→_/21 %_↓_)	108 (95 %_→_/74 %_↓_)	114
Candida glabrata (*p =* 0.0180)			
no	9 (20 %_→_/47 %_↓_)	35 (80 %_→_/22 %_↓_)	44
yes	10 (8 %_→_/53 %_↓_)	121 (92 %_→_/78 %_↓_)	131
Candida krusei (*p =* 0.0000)			
no	38 (48 %_→_/72 %_↓_)	42 (53 %_→_/34 %_↓_)	80
yes	15 (16 %_→_/28 %_↓_)	80 (84 %_→_/66 %_↓_)	95
Candida tropicalis (*p =* 0.043)			
no	157 (96 %_→_/95 %_↓_)	7 (4 %_→_/78 %_↓_)	164
yes	9 (82 %_→_/5 %_↓_)	2 (18 %_→_/22 %_↓_)	11

The correlation of *C. glabrata* occurrence in the samples collected from hand and phone surfaces was also explicit (Table 2). The study population also showed a highly statistically significant correlation between the occurrence of *C. krusei* strains on hand and phone surfaces. Although *C. tropicalis* was relatively infrequently isolated, it can be concluded that its presence on mobile phone surfaces increased the probability of its occurrence on the hands, and vice versa. The p-value did not exceed 0.05, therefore the relationship should be considered statistically significant.

The number of *Candida* colonies present on hand and phone surfaces is described further in this paper. Only individuals who tested positive for the presence of a certain genus of fungi were included in the characteristics. Analysis of the descriptive statistics presented in the table below (Table 3) should take into account the following: the mean number of fungal colonies of the same genera/species was higher in the samples collected from hand surfaces compared with mobile phone surfaces; the median number of fungal colonies was lower than the average, which means that only a few individuals had very low levels of personal hygiene and were positive for large numbers of fungal colonies, for some of which the maximum values of fungi were very high.

**Table 3:** Numbers of *Candida* strains in samples collected from hand and phone surfaces

Number of Candida colonies	*N*	$\overline{x}$	Me	*SD*	*c*_25_	*c*_75_	min.	max.
on the surface of the hand								
*Candida albicans*	146	5.9	3	6.6	2	8	1	30
*Candida glabrata*	156	13.0	8.5	15.7	3	15	1	100
*Candida krusei*	122	12.4	5	19.5	2	12	1	100
*Candida tropicalis*	9	5.2	4	4.6	2	7	1	15
*Candida species*	1	4.0	4	0	4	4	4	4
on the surface of the phone								
*Candida albicans*	114	3.3	2	3.8	1	4	1	20
*Candida glabrata*	131	8.5	5	8.3	2	13	1	40
*Candida krusei*	95	6.3	3	8.0	2	8	1	43
*Candida tropicalis*	11	1.5	1	1.2	1	1	1	5

$\overline{x}$ -mean, Me-mediana, SD- standard deviation

The relationship between the number of colonies of the same fungal strains isolated from the surface of hands and mobile phones was another interesting aspect. For this purpose, a correlation analysis, which only involved individuals who tested positive for the presence of a certain strain both on the hands as well as on their phone, was performed.

Spearman’s rank correlation coefficient was used to analyze the correlation between the numbers of colonies. Although the analyzed correlations were statistically significant, their strength should not be considered high. The strongest correlation was found between the numbers of colonies from the hands and phones for *C. albicans* with R = 0.45 (*p =* 0.0000), whereas the correlations were very weak for the other two analyzed fungal species/genera (the R-value did not exceed 0.30). The R-value was 0.26 (*p =* 0.0039) for *C. glabrata*, and 0.29 (*p =* 0.0081) for *C. krusei*. Therefore, we can conclude that there is a relationship between the number of colonies of the fungi isolated from the mobile phone and the hand surface; however, it was not a close correlation.

The average numbers of fungal colonies on hand and phone surfaces were also compared (Table 4).

**Table 4:** Distribution of the numbers of Candida colonies on hands and phone surfaces

	*N*	$\overline{x}$	Me	*s*	*c*_25_	*c*_75_	min	max
*Candida albicans* (number of colonies)								
on the hand	146	5.9	3	6.6	2	8	1	30
on the phone	114	3.3	2	3.8	1	4	1	20
on phone—on hand (*p =* 0.0000)	108	–2.6	–1	6.1	–4	0	–28	15
*Candida glabrata* (number of colonies)								
on the hand	156	13.0	8.5	15.7	3	15	1	100
on the phone	131	8.5	5	8.3	2	13	1	40
on phone—on hand (*p =* 0.0071)	121	–4.2	–1	14.7	–6	2	–81	29
*Candida krusei* (number of colonies)								
on the hand	122	12.4	5	19.5	2	12	1	100
on the phone	95	6.3	3	8.0	2	8	1	43
on phone—on hand (*p =* 0.0121)	80	–6.8	0	23.1	–10	2	–98	39

$\overline{x}$ -mean, Me-mediana, SD- standard deviation

It was also found that the numbers of *C. albicans* colonies were higher on the hand surface rather on phones (*p =* 0.0000) for most subjects. The mean difference was 2.6 colonies, although the median for the differences was significantly lower, i.e. 1 (the mean is lowered by several cases of extremely large differences between the numbers of colonies on the hands and on phones). Very similar conclusions can be drawn about the number of *C. glabrata* colonies, which were isolated in higher numbers from samples collected from hand surfaces than phones (*p =* 0.0071). In the case of *C. krusei*, on average, nearly 7 colonies less were isolated from the surface of mobile phones, although the median of the differences was 0, which indicates that in at least half of the cases the number of colonies on the hand surface was equal to or lower than the number of colonies on the phone surface (*p =* 0.0121).

Correlation coefficients between the assessments of the health locus of control and the assessment of hand washing habits were also determined. The correlation with the MHLC scale was evaluated for a calculated self-reported average hand washing habit score in various life situations. In order to calculate this measure, it was assumed that the hand washing habit is ‘always’ assessed based on a 3-point score, i.e. frequently – 3 pts., rarely – 1 pts., and never – 0 pts. The mean assessment of hand washing habit was used in the analysis. It could be expected that individuals in support of the opinion that health is determined by an internal control will attach a greater importance to hand washing, and indeed, the only statistically significant correlation was related to these two variables, however, its strength was low, i.e. R =0.19 (*p =* 0.0103*). Other correlations were statistically insignificant, and the correlation coefficients were very close to zero. The R-value was 0.13 (*p =* 0.0766) for individuals who prefer the opinion that health is determined by others, and 0.11 (*p =* 0.1305) for those convinced about the impact of coincidence.

Correlation coefficients between the number of colonies of the individual fungal species and the assessments of the health locus of control were also determined (Table 5). A significant correlation was only shown between the number of *C. albicans* colonies and the assessment of the impact of other people on health. However, this correlation was rather weak and had a negative direction, indicating that the more importance was attached to the impact of others on health, the fewer *C. albicans* colonies were isolated from hand surfaces. Although, it is difficult to substantively explain this correlation.

**Table 5:** Correlations between the numbers of *Candida* colonies isolated from hand surfaces and the health locus of control

	Health locus of control		
	internal control	impact of others	coincidence
hand surface			
*Candia albicans*	–0.10 (*p =* 0.2197)	–0.28 (*p =* 0.0006)	0.03 (*p =* 0.7330)
*Candida glabrata*	–0.02 (*p =* 0.7793)	–0.13 (*p =* 0.1006)	0.07 (*p =* 0.3635)
*Candida krusei*	–0.09 (*p =* 0.3200)	–0.03 (*p =* 0.7050)	0.01 (*p =* 0.8998)
*Candida tropicalis*	–0.57 (*p =* 0.1107)	–0.54 (*p =* 0.1294)	–0.20 (*p =* 0.5981)
telephone surface			
*Candida albicans*	–0.04 (*p =* 0.6533)	–0.12 (*p =* 0.1937)	0.09 (*p =* 0.3149)
*Candida glabrata*	–0.11 (*p =* 0.2028)	–0.09 (*p =* 0.3313)	–0.05 (*p =* 0.5666)
*Candida krusei*	0.01 (*p =* 0.9106)	0.00 (*p =* 0.9644)	0.08 (*p =* 0.4296)
*Candida tropicalis*	0.26 (*p =* 0.4470)	–0.35 (*p =* 0.2970)	–0.53 (*p =* 0.0948)

No significant correlations were found between the numbers of *Candida* colonies on mobile phones and the assessment of health locus of control.

## Discussion

This study aimed to investigate the *Candida* contamination of mobile phones and hands of professors and students of the Medical University Hospital in Białystok, Poland.

In the present study, out of 175 mobile phones, 74.9 % were colonized by *Candida* strains. A number of mobile phones in our study were found to be colonized with potentially pathogenic *Candida.* We found that *Candida albicans*, *Candida glabrata*, and *Candida krusei* were the dominant species in the samples collected from mobile phones and the hands of their owners.

Our findings are consistent with previous reports [7, 12, 13].

Bonassoli et al., [12] examined hand swabs collected from 62 hospital employees and achieved the growth of yeast-like fungi in 59.3 % of subjects. *C. parapsilosis* was the most common species (51 %) isolated from these subjects.

Orsi et al. [13] examined mobile phone microbial contamination among neonatal unit healthcare workers at the teaching Hospital in Rome, Italy. The study participants (*n =* 50), healthcare workers and students, were also asked to complete a questionnaire that included information about age, profession, mobile phone use, and cleaning activity. They found that 86 % of the mobile phones were contaminated with different bacterial strains.

On the other hand, Tambe and Pai [14] reported the prevalence of *Candida* contamination of medical personnel mobile phones lower than our results. They collected swabs from 120 phones belonging to medical personnel and identified contamination in 82.5 % of cases, including 70.8 % of pathogenic bacteria, mainly *Staphylococcus* aureus (54.16 %) and *Micrococcus* (20.83 %). *Candida* strains were isolated in 6.66 % of cases, *Aspergjllus* in 5 %, *Mucor* in 0.8 % and *Trichophyton* in 0.8 %.

The majority of respondents were aware of the fact that the mobile phone surface may be inhabited by microorganisms. Furthermore, as many as 96.6 % of the participants had no knowledge of mobile phone disinfecting machines.

Our findings are consistent with previous reports. Epidemiological studies [15] showed that 30–50 % of physicians and nurses did not disinfect their hands when necessary, and 50–80 % of cosmetologists did not comply with hand hygiene guidelines before or after contact with a patient. Only 52 % of the healthcare workers in the neonatal unit of the teaching Hospital in Rome cleaned their mobile phones [13].

Jarvis [16] emphasized that in the U.S. physicians did not wash their hands more frequently than nurses (14–59 % and 25–45 %, respectively).

In the present study, 81.1 % of participants declared that they had heard that the phone surface may be inhabited by microorganisms. A total of 86.3 % of respondents were convinced of this fact. They found this knowledge mainly on the Internet (43.4 %) and television (33.7 %).

Singh and Purohit [17] emphasized that, as opposed to hands, which may easily be sterilized through the use of disinfectants, mobile phones are difficult to clean. They showed that only 12 % of medical personnel used disinfectants to wipe their phones. Ulger et al. [6] found that only 10 % of medical personnel regularly cleaned their phones, which according to the authors indicates that mobile phones used by health care providers could be a source of hospital-acquired infections. Ramesh et al. [18] found that only 3 % of subjects washed their hands after using their phones, and 53 % never cleaned their phones. In the present study, only 19.4 % of respondents cleaned their phone surfaces.

Mark et al. [5] showed that disinfection with 70 % alcohol significantly reduced the number of isolated microbes, from 98.3 % to 55.2 %. Similarly, Arora et al. [19] showed a 98 % efficacy of 70 % isopropyl alcohol in cleaning mobile phones.

According to Gashaw et al. [20], 70.7 % of respondents claimed that bacteria could be transmitted via mobile phones; however, only 53.4 % of them believed that bacteria could be spread from one patient to another this way. The fact that as many as 29.3 % of respondents kept their mobile phones with medical equipment such as a stethoscope or neurological hammer is alarming. We also found that the majority of participants kept their phones in a shoulder bag (54.9 %), and when moving around in a bag (50.3 %) or a pocket (44 %).

Further research should be conducted in order to investigate the pathogenicity of the fungi isolated from mobile phones, as phone use may play a significant role in the spread of hospital-acquired infections due to the fact that mobile phones, unlike land-line phones, are commonly used in the patient’s environment.

## Conclusions

*C. albicans*, *C. glabrata*, and *C. krusei* were the dominant species in the samples collected from mobile phones and the hands of their owners. The majority of respondents were aware of the fact that the mobile phone surface may be inhabited by microorganisms, mainly bacteria, and would like to have their phones professionally cleaned. A significant correlation was found between the occurrence of fungi in the samples collected from hand and phone surfaces. No significant correlation was found between the preferred health-related behavior and the frequency of washing hands, the way of using a mobile phone, the number of colonies or the isolation frequency for the fungi collected from the surface of the phones and hands of their owners. To reduce hospital-acquired infection, we recommend regular hand washing and a cleaning of mobile phones with 70 % isopropyl alcohol by medical staff during working hours.

## Abbreviations

C*, Candida;* MHLC, the multidemensional health locus of control scale.
